# Hip Fracture Risk Assessment Tools for Adults Aged 80 Years and Older

**DOI:** 10.1001/jamanetworkopen.2024.18612

**Published:** 2024-06-28

**Authors:** Kristine E. Ensrud, John T. Schousboe, Carolyn J. Crandall, William D. Leslie, Howard A. Fink, Peggy M. Cawthon, Deborah M. Kado, Nancy E. Lane, Jane A. Cauley, Lisa Langsetmo

**Affiliations:** 1 Division of Epidemiology and Community Health, School of Public Health, University of Minnesota, Minneapolis; 2 Department of Medicine, University of Minnesota, Minneapolis; 3 Center for Care Delivery and Outcomes Research, Veterans Affairs Health Care System, Minneapolis, Minnesota; 4 HealthPartners Institute, Bloomington, Minnesota; 5 Division of Health Policy and Management, School of Public Health, University of Minnesota, Minneapolis; 6 Department of Medicine, University of California, Los Angeles; 7 Department of Medicine, University of Manitoba, Winnipeg, Manitoba, Canada; 8 Geriatric Research Education and Clinical Center, Veterans Affairs Health Care System, Minneapolis, Minnesota; 9 California Pacific Medical Center Research Institute, San Francisco; 10 Department of Medicine, Stanford University, California; 11 Geriatric Research Education and Clinical Center, Veterans Affairs Health Care System, Palo Alto, California; 12 Department of Internal Medicine, University of California, Davis, Sacramento; 13 Department of Epidemiology, School of Public Health, University of Pittsburgh, Pittsburgh, Pennsylvania

## Abstract

**Importance:**

While adults aged 80 years and older account for 70% of hip fractures in the US, performance of fracture risk assessment tools in this population is uncertain.

**Objective:**

To compare performance of the Fracture Risk Assessment Tool (FRAX), Garvan Fracture Risk Calculator, and femoral neck bone mineral density (FNBMD) alone in 5-year hip fracture prediction.

**Design, Setting and Participants:**

Prognostic analysis of 3 prospective cohort studies including participants attending an index examination (1997 to 2016) at age 80 years or older. Data were analyzed from March 2023 to April 2024.

**Main Outcomes and Measures:**

Participants contacted every 4 or 6 months after index examination to ascertain incident hip fractures and vital status. Predicted 5-year hip fracture probabilities calculated using FRAX and Garvan models incorporating FNBMD and FNBMD alone. Model discrimination assessed by area under receiver operating characteristic curve (AUC). Model calibration assessed by comparing observed vs predicted hip fracture probabilities within predicted risk quintiles.

**Results:**

A total of 8890 participants were included, with a mean (SD) age at index examination of 82.6 (2.7) years; 4906 participants (55.2%) were women, 866 (9.7%) were Black, 7836 (88.1%) were White, and 188 (2.1%) were other races and ethnicities. During 5-year follow-up, 321 women (6.5%) and 123 men (3.1%) experienced a hip fracture; 818 women (16.7%) and 921 men (23.1%) died before hip fracture. Among women, AUC was 0.69 (95% CI, 0.67-0.72) for FRAX, 0.69 (95% CI, 0.66-0.72) for Garvan, and 0.72 (95% CI, 0.69-0.75) for FNBMD alone (FNBMD superior to FRAX, *P* = .01; and Garvan, *P* = .01). Among men, AUC was 0.71 (95% CI, 0.66-0.75) for FRAX, 0.76 (95% CI, 0.72-0.81) for Garvan, and 0.77 (95% CI, 0.72-0.81) for FNBMD alone (*P* < .001 Garvan and FNBMD alone superior to FRAX). Among both sexes, Garvan greatly overestimated hip fracture risk among individuals in upper quintiles of predicted risk, while FRAX modestly underestimated risk among those in intermediate quintiles of predicted risk.

**Conclusions and Relevance:**

In this prognostic study of adults aged 80 years and older, FRAX and Garvan tools incorporating FNBMD compared with FNBMD alone did not improve 5-year hip fracture discrimination. FRAX modestly underpredicted observed hip fracture probability in intermediate-risk individuals. Garvan markedly overpredicted observed hip fracture probability in high-risk individuals. Until better prediction tools are available, clinicians should prioritize consideration of hip BMD, life expectancy, and patient preferences in decision-making regarding drug treatment initiation for hip fracture prevention in late-life adults.

## Key Points

**Question:**

What is the performance of the Fracture Risk Assessment Tool (FRAX), Garvan Fracture Risk Calculator, and femoral neck bone mineral density (FNBMD) alone in 5-year hip fracture prediction in late-life adults?

**Findings:**

Among 8890 adults aged 80 years and older, FNBMD alone discriminated hip fracture risk at least as well as FRAX or Garvan tools incorporating FNBMD. FRAX was well calibrated, but Garvan markedly overpredicted observed hip fracture probability in high-risk individuals.

**Meaning:**

These findings support the relative importance of FNBMD measurement compared with reliance on FRAX or Garvan tools in hip fracture risk prediction in late-life adults.

## Teaser

This prognostic study compares the performance of different risk assessment tools for 5-year hip fracture among adults aged 80 years and older.

## Introduction

Adults 80 years and older account for 70% of hip fractures in the US.^1,2^ About 20% to 30% of patients with hip fracture die within the following year,^1,3^ and among survivors, impaired mobility, disability, and institutionalization are common.^4,5^ Hip fractures account for 72% of fracture-related US health care expenditures.^6^ Thus, identification of late-life adults at high hip fracture risk is essential.

Low bone mineral density (BMD) is a strong predictor of hip fracture, although the gradient of risk declines with advancing age.^7^ Because clinical risk factors contribute to fracture risk beyond that from BMD alone,^8^ tools including the Fracture Risk Assessment Tool (FRAX)^9^ and Garvan fracture risk calculator^10^ have been developed that estimate a patient’s absolute risk of hip fracture. Several US guidelines^11,12,13,14^ endorse use of FRAX in clinical decision-making regarding whether to initiate drug treatment for fracture prevention. However, few data are available on tool performance in predicting hip fractures in adults 80 years and older. Applicability of current tools may be suboptimal in hip fracture risk prediction in late life for several reasons.^15^ Models should fully account for competing mortality risk and use a relevant prediction timeframe (eg, 5 years). Additionally, models may need to account for relevant nonskeletal risk factors (eg, frailty) increasingly prevalent later in life. To compare the performance of FRAX and Garvan tools incorporating femoral neck BMD (FNBMD) and FNBMD alone in hip fracture risk prediction in US late-life adults, we used a unique longitudinal multicohort dataset of 8890 community-dwelling adults aged 80 years and older who were subsequently followed up for 5 years for incident hip fracture and vital status.

## Methods

### Participants

This prognostic study included community-dwelling adults in 3 prospective cohort studies at least age 80 years at an in-person index examination (1997 to 2016) including FNBMD measurement and clinical risk factor assessment: year (Y) 10 or Y16 examination of Study of Osteoporotic Fractures (SOF); Y3, Y5 or Y6, Y8, or Y10 examination of Health, Aging and Body Composition Study (HealthABC); and Y5, Y7, or Y14 examination of Osteoporotic Fractures in Men Study (MrOS) (eTable 1 and eFigure 1 in Supplement 1). Participants were entered into the analysis based on the examination when they first reached age 80 years (index examination). Race was self-reported by the participant and included in this article as a characteristic for the reader’s information. This study was determined by the University of Minnesota to be exempt from the need for institutional review board review and the need for informed consent because data were deidentified. We followed Transparent Reporting of a Multivariable Prediction Model for Individual Prognosis or Diagnosis (TRIPOD) reporting guidelines.

### Ascertainment of Clinical Fractures and Mortality

Participants were contacted every 4 (SOF, MrOS) or 6 (HealthABC) months after the baseline examination to ascertain vital status and clinical fractures. Over 95% of these contacts in each cohort were completed in active participants. Self-reported hip fractures were confirmed by radiographic reports. Deaths were verified with death certificates. Participants in this analysis were followed up to a maximum of 5 years after the index examination until an event (hip fracture or death) or censoring (mean [SD] follow-up to hip fracture or censoring, 4.4 [1.2] years in both sexes). A 5-year follow-up timeframe was selected because of trials^16^ demonstrating reduced hip fracture risk with osteoporosis drug treatment during this period and noting that average life expectancy is less than 10 years in US women and men after age 80.

### Calculation of 5-Year Hip Fracture Probabilities Using FRAX and Garvan Tools

The publicly available FRAX tool^9^ can be used free of charge by entering clinical risk factors and FNBMD into the web-based tool, which calculates a 10-year predicted probability of hip fracture. The FRAX algorithm is also programmed directly into many bone densitometers. However, the specific algorithms developed by the FRAX center (University of Sheffield, UK) are not publicly available. The US FRAX 10-year predicted hip fracture probabilities for all participants in this study were calculated by the FRAX center using models with FNBMD (version 3.12, 2017) based on values of clinical risk factors and FNBMD at the index examination. Previous fracture in each cohort was identified by participant self-report of a fracture since age 50 at baseline or subsequent confirmed incident clinical fracture. Information about parental history of hip fracture was obtained at baseline in SOF and MrOS, but in HealthABC, these data were not available. Thus, we used the default value (no) for this characteristic for HealthABC participants in the primary analysis, as recommended by the FRAX website. Participants were asked to bring medications used within the preceding month to clinic examinations in all cohorts. Medications were recorded and data were matched by codes to a database.^17^ SOF and MrOS participants in were asked about a diagnosis of rheumatoid arthritis at the index examination; rheumatoid arthritis in HealthABC participants was identified at the index examination by use of methotrexate for arthritis treatment. Other secondary causes of osteoporosis were not considered because when FNBMD is entered into the FRAX tool, no weight is accorded by other secondary causes.^18^ FNBMD in all participants was measured using dual-energy x-ray absorptiometry (Hologic, Inc) at the index examination. FRAX 10-year probabilities were multiplied by 0.5 to convert them to 5-year probabilities.^19,20^

The Garvan 5-year hip fracture probability incorporating FNBMD was calculated for each participant using the published algorithm.^21^ Data on Garvan clinical risk factors were obtained at the index examination. Number of previous fractures was determined using self-report of fractures since age 50 years at the baseline examination and subsequent confirmed incident clinical fractures. Number of falls in the past year was assessed by questionnaire.

### Statistical Analysis

Analyses were stratified by sex. Participant characteristics stratified by event type (hip fracture, death before hip fracture, and survival at 5 years free of hip fracture) were compared.

Tool performance was assessed by evaluating model discrimination (ability to distinguish between participants who did vs did not experience a hip fracture during the 5-year follow-up period) and calibration (agreement between actual observed and model predicted 5-year hip fracture probabilities). We used receiver operating characteristic (ROC) curves to compare model performance in hip fracture discrimination. We calculated area under the ROC curve (AUC) for the FRAX with FNBMD model, Garvan with FNBMD model, and FNBMD alone.

Calibration of FRAX with FNBMD and Garvan with FNBMD models was assessed by comparing the 5-year predicted vs observed hip fracture probability within quintiles of predicted risk. Observed 5-year hip fracture probabilities in the primary analysis were calculated using the cumulative incidence function that accounts for competing mortality risk.^22^ Multivariable Fine-Gray competing risk models^23^ were used to determine subdistribution hazard ratios of hip fracture for each individual component of FRAX and Garvan algorithms.

While we included participants receiving bisphosphonates in the primary analyses, we conducted a sensitivity analysis excluding bisphosphonate users. We also performed sensitivity analyses to assess whether missing data on parental hip fracture in HealthABC impacted results (ie, analyses excluding HealthABC participants and analyses using imputed values for parental hip fracture in HealthABC participants). Finally, since the Garvan model was constructed without considering competing mortality risk, we assessed the calibration using estimates of the observed hip fracture probability calculated using 1−KM, where KM is the traditional Kaplan-Meier survival function.

Statistical significance was defined as *P* < .05 when the null hypothesis test was performed and CIs excluding the null value when 95% CIs were calculated. Analyses were completed using Stata MP version 17.0 (Stata Corp). Data were analyzed from March 2023 to April 2024.

## Results

A total of 8890 participants were included. Mean (SD) age at the index examination was 82.6 (2.7) years in the cohort of 4906 (55.2%) women and 82.7 (2.7) years in the cohort of 3984 (44.8%) men; 866 participants (9.7%) were Black, 7836 (88.1%) were White, and 188 (2.1%) were other races and ethnicities (Asian, Hispanic, Latino, and Native American or Hawaiian or Pacific Islander) (Table 1; eTable 2 and eTable 3 in Supplement 1). A total of 2493 women (50.8%) and 1135 men (28.5%) reported at least 1 prior fracture since age 50 years. A total of 1649 women (33.6%) and 1357 men (34.0%) had fallen in the past year. Mean (SD) femoral neck BMD T score was −1.9 (1.0) in women and −0.8 (1.1) in men. Mean (SD) estimated 5-year hip fracture probability ranged from 4.4% (4.6%; FRAX tool) to 6.7% (5.3%; FNBMD alone) to 16.8% (21.2%; Garvan calculator) in women and 2.0% (2.2%; FRAX tool) to 3.1% (3.5%; FNBMD alone) to 5.6% (8.9%; Garvan calculator) in men.

**Table 1.** Characteristics of Participants by Sex

Characteristic	Participants, No. (%)	
	Women (n = 4906)	Men (n = 3984)
Age, mean (SD), y	82.6 (2.7)	82.7 (2.7)
Race and ethnicity		
Black	545 (11.1)	321 (8.1)
White	4361 (88.9)	3475 (87.2)
Other^a^	0	188 (4.7)
Weight, kg, mean (SD)	65.4 (12.7)	79.4 (12.7)
Body mass index, mean (SD)^b^	26.6 (4.8)	26.8 (3.8)
No. of fractures since age 50 y		
0	2413 (49.2)	2849 (71.5)
1	974 (19.9)	791 (19.9)
2	950 (19.4)	214 (5.4)
≥3	569 (11.6)	130 (3.3)
No. of falls in past year		
0	3257 (66.4)	2627 (65.9)
1	956 (19.5)	703 (17.6)
2	391 (8.0)	499 (12.5)
≥3	302 (6.2)	155 (3.9)
Parent fractured hip^c^	494 (10.1)	431 (10.8)
Current smoker	152 (3.1)	74 (1.9)
Oral glucocorticoid use	165 (3.4)	108 (2.7)
Rheumatoid arthritis	597 (12.2)	213 (5.3)
Alcohol intake ≥3 drinks/d	38 (0.8)	145 (3.6)
Bisphosphonate use	497 (10.2)	174 (4.4)
Femoral neck BMD T score, mean (SD)^d^	−1.9 (1.0)	−0.8 (1.1)
5-y probability of hip fracture from FRAX w/BMD model, mean (SD), %^c^	4.4 (4.6)	2.0 (2.2)
5-y probability of hip fracture from Garvan w/BMD model, mean (SD), %	16.8 (21.2)	5.6 (8.9)
5-y probability of hip fracture from femoral neck BMD, mean (SD), %	6.7 (5.3)	3.1 (3.5)

Abbreviation: BMD, bone mineral density.

^a^ Other includes Asian, Hispanic, Latino, American Indian or Alaska Native, or Hawaiian or Pacific Islander.

^b^ Body mass index is calculated as weight in kilograms divided by height in meters squared.

^c^ Assuming no parental history of hip fracture for all Health ABC participants.

^d^ BMD T scores were calculated using the National Health and Nutrition Examination Survey young white female reference database.

During the 5 years after the index examination, 321 women (6.5%) and 123 men (3.1%) experienced a hip fracture, and 818 women (16.7%) and 921 men (23.1%) died before hip fracture. Among women and men, characteristics of participants with incident hip fracture, those who died before experiencing hip fracture, and those who survived 5 years free of hip fracture are shown in Table 2.

**Table 2.** Characteristics of Participants Stratified by Type of Event

Characteristic	Participants, No. (%)			*P* value^a^
	Hip fracture	Death before hip fracture	Survival at 5 y free of hip fracture	
**Women**				
Total No.	321	818	3767	NA
Age, mean (SD), y	83.4 (3.3)	83.6 (3.6)	82.4 (2.4)	<.001
Race and ethnicity				
Black	15 (4.7)	93 (11.4)	437 (11.6)	.001
White	306 (95.3)	725 (88.6)	3330 (88.4)	
Other^b^	0	0	0	
Weight, mean (SD), kg	60.9 (11.1)	63.2 (13.3)	66.3 (12.5)	<.001
Body mass index, mean (SD)^c^	24.7 (4.4)	25.9 (5.0)	26.9 (4.8)	<.001
No. of fractures since age 50 y				
0	112 (34.9)	362 (44.3)	1939 (51.5)	<.001
1	66 (20.6)	176 (21.5)	732 (19.4)	
2	82 (25.5)	173 (21.1)	695 (18.4)	
≥3	61 (19.0)	107 (13.1)	401 (10.6)	
No. of falls in past year				
0	197 (61.4)	521 (63.7)	2539 (67.4)	.06
1	72 (22.4)	156 (19.1)	728 (19.3)	
2	31 (9.7)	74 (9.0)	286 (7.6)	
≥3	21 (6.5)	67 (8.2)	214 (5.7)	
Parent fractured hip^d^	40 (12.5)	79 (9.7)	375 (10.0)	.38
Current smoker	12 (3.7)	42 (5.1)	98 (2.6)	.12
Oral glucocorticoid use	16 (5.0)	45 (5.5)	104 (2.8)	<.001
Rheumatoid arthritis	43 (13.4)	125 (15.3)	429 (11.4)	.02
Alcohol intake ≥3 drinks/d	3 (0.9)	10 (1.2)	25 (0.7)	.43
Femoral neck BMD T score, mean (SD)	−2.6 (0.8)	−2.1 (1.0)	−1.8 (1.0)	<.001
5-y probability of hip fracture from FRAX w/BMD model, mean (SD), %^d^	6.9 (5.9)	4.8 (5.0)	4.1 (4.3)	<.001
5-y probability of hip fracture from Garvan w/BMD model, mean (SD), %	30.1 (28.1)	20.8 (24.2)	14.8 (19.2)	<.001
5-y probability of hip fracture from femoral neck BMD, mean (SD), %	10.7 (6.6)	7.8 (6.5)	6.2 (4.7)	<.001
**Men**				
Total No.	123	921	2940	NA
Age, mean (SD), y	83.6 (3.2)	83.4 (3.2)	82.4 (2.4)	<.001
Race and ethnicity				
Black	8 (6.5)	96 (10.4)	217 (7.4)	.003
White	114 (92.7)	790 (85.8)	2571 (87.4)	
Other^b^	1 (0.8)	35 (3.8)	152 (5.2)	
Weight, kg, mean (SD)	76.6 (11.1)	77.4 (13.3)	80.1 (12.4)	<.001
Body mass index, mean (SD)^c^	25.7 (3.4)	26.2 (3.9)	27.0 (3.7)	<.001
No. of fractures since age 50 y				
0	74 (60.2)	642 (69.7)	2133 (72.6)	<.001
1	27 (22.0)	195 (21.2)	569 (19.4)	
2	11 (8.9)	56 (6.1)	147 (5.0)	
≥3	11 (8.9)	28 (3.0)	91 (3.1)	
No. of falls in past year				
0	65 (52.8)	569 (61.8)	1993 (67.8)	<.001
1	21 (17.1)	159 (17.3)	523 (17.8)	
2	28 (22.8)	141 (15.3)	330 (11.2)	
≥3	9 (7.3)	52 (5.6)	94 (3.2)	
Parent fractured hip^d^	15 (12.2)	91 (9.9)	325 (11.1)	.67
Current smoker	6 (4.9)	28 (3.0)	40 (1.4)	.01
Oral glucocorticoid use	3 (2.4)	40 (4.3)	65 (2.2)	.003
Rheumatoid arthritis	11 (8.9)	58 (6.3)	144 (4.9)	.02
Alcohol intake ≥3 drinks/d	2 (1.6)	28 (3.0)	115 (3.9)	.27
Femoral neck BMD T score, mean (SD)	−1.8 (1.0)	−0.9 (1.1)	−0.8 (1.1)	<.001
5-y probability of hip fracture from FRAX w/BMD model, mean (SD), %^d^	3.4 (3.2)	2.1 (2.1)	2.0 (2.1)	<.001
5-y probability of hip fracture from Garvan w/BMD model, mean (SD), %	15.7 (17.5)	7.0 (10.7)	4.7 (7.3)	<.001
5-y probability of hip fracture from femoral neck BMD, mean (SD), %	6.8 (6.6)	3.5 (3.6)	2.9 (3.1)	<.001

Abbreviations: BMD, bone mineral density; FRAX, Fracture Risk Assessement Tool; NA, not applicable.

^a^ Participant characteristics stratified by event type (hip fracture, death before hip fracture, or survival at 5 years free of hip fracture) were compared using ANOVA for normally distributed continuous variables, χ^2^ tests for categorical variables, and Kruskal-Wallis nonparametric tests for skewed variables.

^b^ Other includes Asian, Hispanic, Latino, and American Indian or Alaska Native, or Hawaiian or Pacific Islander.

^c^ Body mass index is calculated as weight in kilograms divided by height in meters squared.

^d^ Assuming no parental history of hip fracture for all Health ABC participants.

**Figure 1.**
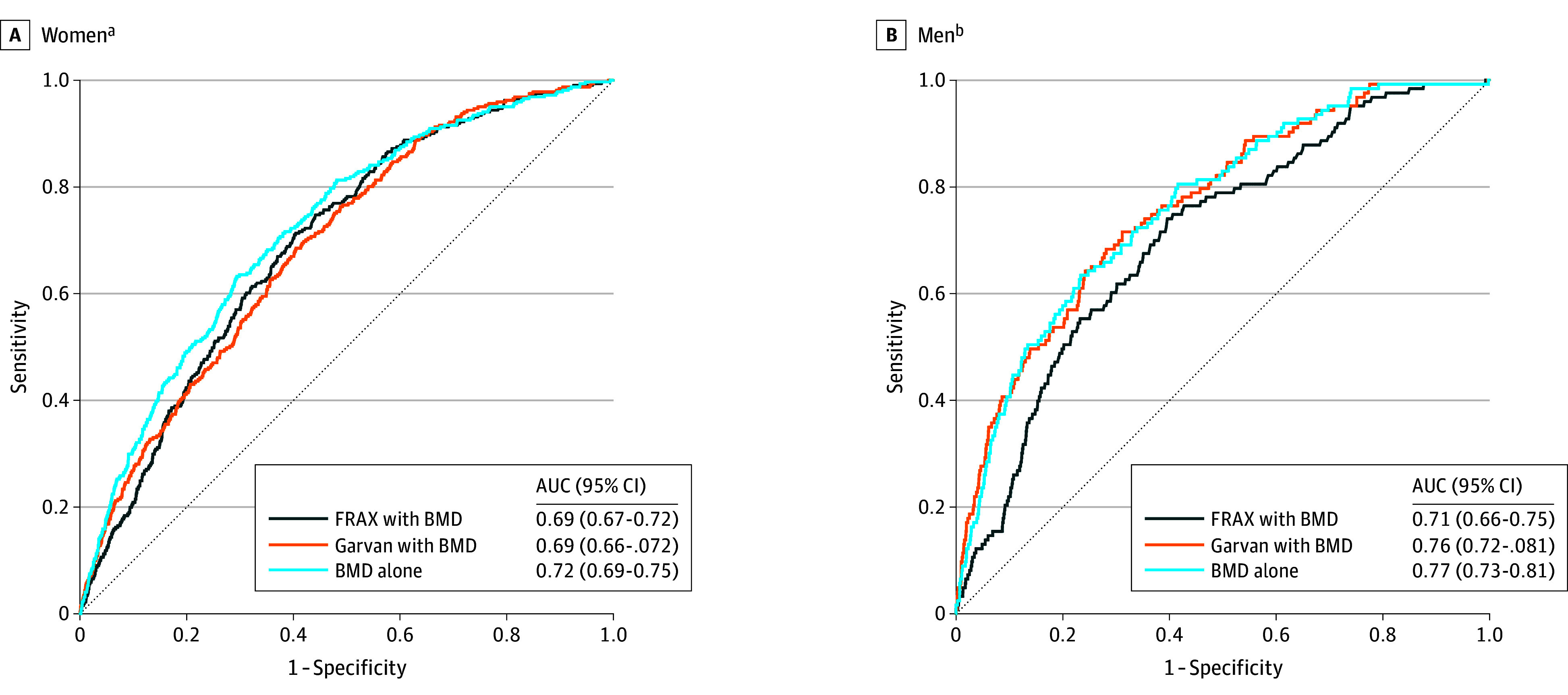
Receiver Operating Characteristic Curves for Hip Fracture Prediction in Women and Men Bone mineral density (BMD) measured at the femoral neck. AUC indicates area under the receiver operating characteristic curve. FRAX indicates Fracture Risk Assessment Tool. ^a^*P* = .007 for femoral neck BMD alone vs FRAX, .01 for femoral neck BMD alone vs Garvan, and .71 for FRAX vs Garvan. ^b^*P* < .001 for femoral neck BMD alone vs FRAX, .81 for femoral neck BMD alone vs Garvan, and <.001 for FRAX vs Garvan.

In terms of discriminating between participants who did vs did not experience a hip fracture during the 5-year follow-up, ROC curves in women were essentially superimposed for FRAX and Garvan (Figure 1). The AUC was 0.69 (95% CI, 0.67-0.72) for FRAX and 0.69 (95% CI, 0.66-0.72) for Garvan (*P* = .71). FNBMD alone with an AUC 0.72 (95% CI, 0.69-0.75) was superior to both FRAX (*P* = .01) and Garvan (*P* = .01) in hip fracture discrimination in women. Among men, ROC curves for hip fracture prediction were nearly identical for Garvan and FNBMD alone (Figure 1). The AUC was 0.76 (95% CI, 0.72-0.81) for Garvan and 0.77 (95% CI, 0.73-0.81) for FNBMD alone (*P* = .81). In men, the FRAX with an AUC of 0.71 (95% CI, 0.66-0.75) was inferior to both Garvan (*P* < .001) and FNBMD alone (*P* < .001) in hip fracture discrimination.

**Figure 2.**
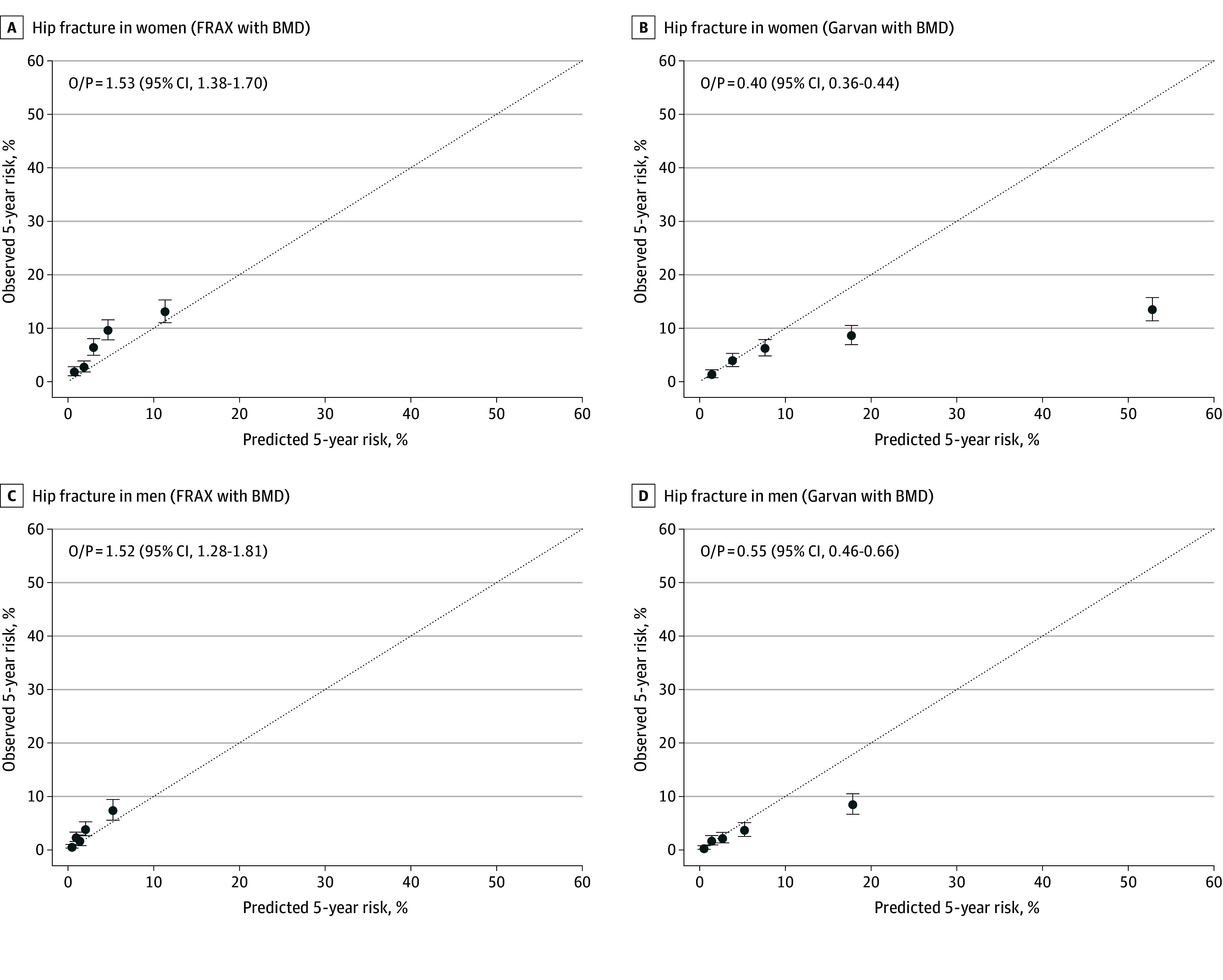
Observed vs Predicted 5-Year Absolute Probability of Hip Fracture for Fracture Risk Assessment Tool (FRAX) and Garvan Models Stratified by Quintile of Predicted Risk in Women and Men Observed probability of hip fracture was calculated using cumulative incidence function. Dotted line indicates perfect calibration between predicted and observed probabilities. BMD indicates bone mineral density; O/P, mean observed probability/predicted probability for overall cohort.

With respect to model calibration (agreement between actual observed vs predicted 5-year hip fracture probability within quintiles of predicted risk), FRAX underpredicted 5-year hip fracture probability among individuals in intermediate risk quintiles in both sexes (Figure 2A). In contrast, Garvan greatly overestimated 5-year hip fracture probability among individuals in upper quintiles of predicted risk in both sexes (Figure 2B).

In an analysis estimating associations of individual components of FRAX with hip fracture risk accounting for death as a competing risk, lower FNBMD was independently associated with an increased risk of hip fracture in both sexes (Table 3). Older age appeared to be associated with a higher risk of hip fracture in both sexes, but the association was lesser in women with CIs that slightly overlapped 1.0. Prior fracture also appeared to be a factor associated with risk in both sexes, but the association was significant only in women. Among men, but not women, rheumatoid arthritis was associated with a higher risk of hip fracture. In a similar analysis examining Garvan individual components, lower FNBMD and multiple prior fractures were independently associated with increased risks of hip fracture in both sexes. Older age was moderately associated with a higher risk of hip fracture in men, but more modestly associated with risk of hip fracture among women with CIs slightly overlapping 1.0. Having 2 falls in the past year was associated with a higher risk of hip fracture in men, but number of falls was not significantly associated with risk of hip fracture among women.

**Table 3.** Subdistribution Models for Associations of Individual Components of Fracture Risk Assessment Tool (FRAX) and Garvan Tools With Risk of Hip Fracture

Component	Subdistribution HR (95% CI) of hip fracture	
	Women (n = 4906)	Men (n = 3984)
FRAX		
Age (per 5-y increase)	1.17 (0.98-1.39)	1.61 (1.20-2.15)^a^
BMI (per 1 increase)^b^	0.97 (0.94-1.00)	1.00 (0.95-1.05)
Prior fracture	1.35 (1.06-1.72)^a^	1.32 (0.92-1.88)
Parental hip fracture^c^	1.10 (0.78-1.55)	1.03 (0.59-1.80)
Current smoker	1.06 (0.60-1.89)	1.91 (0.74-4.94)
Oral glucocorticoid use	1.36 (0.81-2.27)	0.73 (0.23-2.33)
Rheumatoid arthritis	1.06 (0.76-1.47)	2.16 (1.15-4.05)^a^
Alcohol intake ≥3 drinks/d	1.26 (0.41-3.82)	0.63 (0.15-2.61)
Femoral neck BMD T score (per 1-SD decrease)	2.08 (1.78-2.43)^a^	2.62 (2.10-3.26)^a^
Garvan		
Age (per 5-y increase)	1.16 (0.98-1.38)	1.55 (1.16-2.08)^a^
Prior fracture		
None	1 [Reference]	1 [Reference]
1	1.14 (0.84-1.55)	1.08 (0.70-1.67)
2	1.44 (1.07-1.95)^a^	1.34 (0.72-2.50)
≥3	1.48 (1.06-2.06)^a^	2.30 (1.15-4.59)^a^
Falls in past year		
None	1 [Reference]	1 [Reference]
1	1.16 (0.88-1.52)	1.06 (0.64-1.75)
2	1.30 (0.88-1.91)	2.21 (1.41-3.46)^a^
≥3	1.12 (0.71-1.79)	1.69 (0.79-3.62)
Femoral neck BMD T score (per 1-SD decrease)	2.21 (1.92-2.53)^a^	2.63 (2.15-3.22)^a^

Abbreviations: BMD, bone mineral density; BMI, body mass index; HR, hazard ratio.

^a^ The 95% CI for this association does not contain 1.00.

^b^ Body mass index is calculated as weight in kilograms divided by height in meters squared.

^c^ Assuming no parental history of hip fracture for all Health ABC participants.

Findings were similar in sensitivity analyses limited to participants in SOF and MrOS and analyses substituting FRAX probabilities generated using imputed values for parental hip fracture history in HealthABC participants. Results were also similar in secondary analyses excluding participants taking bisphosphonates at the index examination. Finally, results regarding Garvan calibration were nearly identical when the observed hip fracture probability was calculated using the traditional KM method (eFigure 2 in Supplement 1).

## Discussion

Hip fracture discrimination at 5 years in this large prognostic study of community-dwelling US adults 80 years and older was fair to acceptable with FRAX and Garvan tools incorporating FNBMD but was inferior or similar to the discrimination of FNBMD alone. Garvan markedly overpredicted hip fracture probability in high-risk individuals, while FRAX modestly underestimated hip fracture probability in intermediate-risk individuals.

In our study of US late-life adults (mean 83 years), FRAX and Garvan tools had lower discriminative ability for hip fracture prediction than that reported for younger populations. A systematic review concluded that AUCs for hip fracture were significantly lower in studies with higher mean age.^24^ Specifically, the AUC for 5-year hip fracture discrimination in our study for FRAX incorporating FNBMD was 0.69 in women and 0.71 in men. In contrast, the original derivation and validation study of FRAX with FNBMD^8^ reported a mean AUC for 10-year hip fracture prediction of 0.78 in the pooled 9 derivation cohorts (mean age 65 years) and 0.74 in the pooled 11 validation cohorts (mean age 63 years). Of note, the risk gradient for hip fracture prediction per SD change in the FRAX with FNBMD risk score in this study declined with advancing age, with a 4.2-fold increase per 1-SD increment among individuals age 50 years compared with a 2.0-fold increase per 1-SD increment among adults age 90 years. Better hip fracture discrimination with FRAX incorporating FNBMD than we observed has also been reported in single study populations of North American adults with lower mean age, including the overall SOF cohort (mean age 71 years),^25^ the overall MrOS cohort (mean age 74 years),^26^ the Canadian Multicenter Osteoporosis Study (CaMos) (mean age 66 years),^27^ and the Manitoba BMD registry^28^ (mean age in women 66 years, and in men, 68 years).

Hip fracture discrimination at 5 years of Garvan with FNBMD in our study was fair in women (AUC, 0.69) and good in men (AUC, 0.76). In contrast, the original derivation study of Garvan incorporating FNBMD^21^ in 1208 women and 740 men aged 60 years and older (mean age not provided) in the Australian Dubbo Osteoporosis Epidemiology Study reported excellent hip fracture discrimination (AUC for both sexes, 0.85). Similarly, external validation studies conducted in younger Canadian populations also found excellent hip fracture discrimination of the Garvan tool with BMD.^29,30^

The usefulness of a risk prediction tool in the busy clinical practice setting depends not only on its diagnostic accuracy, but also on its ease of use. In our study, FNBMD alone discriminated between women and men aged 80 years and older who did vs did not experience a hip fracture in the subsequent 5 years at least as well as more complex Garvan and FRAX tools incorporating FNBMD. A previous systematic review^31^ concluded that simple tools with fewer risk factors often perform as well as more complex instruments in discriminating incident osteoporotic fracture, including hip fracture. Additionally, previous external validation studies in US adults aged 65 years and older using data from SOF^25^ and MrOS^32^ reported that a simple model based on age and FNBMD alone predicted 10-year hip fracture probability as well as FRAX with FNBMD. We found that lower FNBMD was an independent factor associated with risk for hip fracture in late-life women and men after accounting for competing mortality risk. However, our results suggest that increasing age is not as greatly associated with hip fracture in late-life adults compared with younger older populations, especially among women. This finding likely reflects a wider age range of the younger older populations and the higher competing mortality risk before hip fracture in late life.

FRAX modestly underpredicted 5-year hip fracture probability in intermediate-risk individuals. FRAX was developed using multiple international cohorts of adults aged 40 to 90 years with a combined sample size of 60 000 individuals in the pooled development cohorts. Country-specific by design, FRAX considers risk of death in calculating fracture probabilities, generates risk estimates of fracture and death using Poisson regression, and is calibrated using national age- and sex-stratified data on hip fracture and mortality for the target population. These model features likely yielded better calibration of hip fracture probability in our study of late-life US adults than that found with the Garvan algorithm.

Garvan markedly overestimated hip fracture probability among individuals at highest risk whether the observed probability was calculated with or without accounting for competing mortality risk. It was developed using a single cohort of 1768 adults aged 60 years and older residing in Dubbo, Australia. Furthermore, the Garvan model assumes that hip fracture hazards rise exponentially with advancing age. This assumption and sparse data in participants aged 80 years and older likely contributed to poorly calibrated predicted risk estimates in our study population.

Our results support the relative importance of hip BMD measurement compared with reliance on FRAX or Garvan tools in clinical decision-making regarding initiation of drug treatment for hip fracture prevention in community-dwelling adults 80 years and older. Previous research using SOF data^33^ found that women aged 80 years and older with BMD-defined osteoporosis (femoral neck or total hip BMD T score −2.5 or less), including those with more comorbidities or limited life expectancy, have a high 5-year hip fracture probability despite accounting for competing mortality. In contrast, competing mortality far outweighed hip fracture probability among women aged 80 years and older without osteoporosis, especially among those with more comorbidities or poorer prognosis. While a better evidence base is clearly required to quantify the absolute benefits vs harms of osteoporosis drug treatment in late-life adults, our findings suggest that hip BMD measurement and estimated life expectancy^34^ are paramount to consider along with individual patient values and preferences.

Study strengths include the large cohort of well-characterized community-dwelling adults aged 80 years and older and nearly complete participant follow-up for vital status and hip fracture confirmed with radiographic reports. Additionally, we calculated observed hip fracture probability accounting for competing mortality risk.

### Limitations

Our cohort comprised predominantly non-Hispanic White individuals. While this population currently accounts for most US hip fractures, our results require confirmation in other racial and ethnic groups. Our results do not apply to late-life adults residing in institutions. However, hip fracture risk prediction is of utmost relevance to independent-living individuals as they are at highest risk of functional decline after hip fracture. We did not evaluate the performance of the Qfracture tool^35^ because it does not incorporate BMD into its hip fracture risk prediction. In addition, a recent study^36^ found that Qfracture and a modified model using the same covariates that accounted for competing mortality risk performed poorly in late-life adults, with discriminative ability only a little better than chance alone and substantial overprediction of observed hip fracture probabilities across deciles of predicted 10-year risk. Finally, precision of estimated associations of low prevalence risk factors with hip fracture was limited, resulting in wide CIs around the point estimates of associations, especially in men.

## Conclusions

In our large study of community-dwelling US adults aged 80 years and older, FNBMD alone performed at least as well in 5-year hip fracture discrimination as more complex FRAX and Garvan tools incorporating FNBMD. FRAX modestly underestimated hip fracture probability in intermediate-risk individuals, while Garvan markedly overestimated hip fracture probability in high-risk individuals. Until better risk prediction tools are available, clinicians should prioritize consideration of hip BMD, life expectancy, and patient preferences in decision-making regarding drug treatment initiation for hip fracture prevention in late-life adults.

## Notes

Supplement 1. **eTable 1. **Cohort Study Information **eTable 2. **Characteristics of Women by Cohort **eTable 3. **Characteristics of Men by Cohort **eFigure 1. **Participant Flow Diagram **eFigure 2. **Observed vs Predicted 5-Year Absolute Probability of Hip Fracture Stratified by Quintile of Predicted Risk for the Garvan Tool in Women and Men

Supplement 2.
Data Sharing Statement

## References

[1] Brauer CA, Coca-Perraillon M, Cutler DM, Rosen AB. Incidence and mortality of hip fractures in the United States. JAMA. 2009;302(14):1573-1579. doi:10.1001/jama.2009.1462 19826027 PMC4410861

[2] Lewiecki EM, Wright NC, Curtis JR, . Hip fracture trends in the United States, 2002 to 2015. Osteoporos Int. 2018;29(3):717-722. doi:10.1007/s00198-017-4345-0 29282482

[3] Abrahamsen B, van Staa T, Ariely R, Olson M, Cooper C. Excess mortality following hip fracture: a systematic epidemiological review. Osteoporos Int. 2009;20(10):1633-1650. doi:10.1007/s00198-009-0920-3 19421703

[4] Dyer SM, Crotty M, Fairhall N, ; Fragility Fracture Network (FFN) Rehabilitation Research Special Interest Group. A critical review of the long-term disability outcomes following hip fracture. BMC Geriatr. 2016;16(1):158. doi:10.1186/s12877-016-0332-0 27590604 PMC5010762

[5] Tajeu GS, Delzell E, Smith W, . Death, debility, and destitution following hip fracture. J Gerontol A Biol Sci Med Sci. 2014;69(3):346-353. doi:10.1093/gerona/glt105 23873945 PMC3976138

[6] Burge R, Dawson-Hughes B, Solomon DH, Wong JB, King A, Tosteson A. Incidence and economic burden of osteoporosis-related fractures in the United States, 2005-2025. J Bone Miner Res. 2007;22(3):465-475. doi:10.1359/jbmr.061113 17144789

[7] Johnell O, Kanis JA, Oden A, . Predictive value of BMD for hip and other fractures. J Bone Miner Res. 2005;20(7):1185-1194. doi:10.1359/JBMR.050304 15940371

[8] Kanis JA, Oden A, Johnell O, . The use of clinical risk factors enhances the performance of BMD in the prediction of hip and osteoporotic fractures in men and women. Osteoporos Int. 2007;18(8):1033-1046. doi:10.1007/s00198-007-0343-y 17323110

[9] Centre for Metabolic Bone Diseases UoSU. FRAX WHO fracture risk assessment tool. 2020. Accessed December 20, 2023. https://www.sheffield.ac.uk/FRAX/

[10] Garvan Institute of Medical Research. Bone FRACTURE RISK CALCULATOR. 2023. Accessed December 20, 2023. https://www.garvan.org.au/bone-fracture-risk

[11] Camacho PM, Petak SM, Binkley N, . American Association of Clinical Endocrinologists/American College of Endocrinology clinical practice guidelines for the diagnosis and treatment of postmenopausal osteoporosis—2020 update. Endocr Pract. 2020;26(suppl 1):1-46. doi:10.4158/GL-2020-0524SUPPL 32427503

[12] Eastell R, Rosen CJ, Black DM, Cheung AM, Murad MH, Shoback D. Pharmacological management of osteoporosis in postmenopausal women: An Endocrine Society* clinical practice guideline. J Clin Endocrinol Metab. 2019;104(5):1595-1622. doi:10.1210/jc.2019-00221 30907953

[13] LeBoff MS, Greenspan SL, Insogna KL, . The clinician’s guide to prevention and treatment of osteoporosis. Osteoporos Int. 2022;33(10):2049-2102. doi:10.1007/s00198-021-05900-y 35478046 PMC9546973

[14] Watts NB, Adler RA, Bilezikian JP, ; Endocrine Society. Osteoporosis in men: an Endocrine Society clinical practice guideline. J Clin Endocrinol Metab. 2012;97(6):1802-1822. doi:10.1210/jc.2011-3045 22675062

[15] Berry SD, Kiel DP, Colón-Emeric C. Hip fractures in older adults in 2019. JAMA. 2019;321(22):2231-2232. doi:10.1001/jama.2019.5453 31074763 PMC6800121

[16] Ayers C, Kansagara D, Lazur B, Fu R, Kwon A, Harrod C. Effectiveness and safety of treatments to prevent fractures in people with low bone mass or primary osteoporosis: a living systematic review and network meta-analysis for the American College of Physicians. Ann Intern Med. 2023;176(2):182-195. doi:10.7326/M22-0684 36592455

[17] Pahor M, Chrischilles EA, Guralnik JM, Brown SL, Wallace RB, Carbonin P. Drug data coding and analysis in epidemiologic studies. Eur J Epidemiol. 1994;10(4):405-411. doi:10.1007/BF01719664 7843344

[18] Kanis JA, Oden A, Johansson H, Borgström F, Ström O, McCloskey E. FRAX and its applications to clinical practice. Bone. 2009;44(5):734-743. doi:10.1016/j.bone.2009.01.373 19195497

[19] Dagan N, Cohen-Stavi C, Leventer-Roberts M, Balicer RD. External validation and comparison of three prediction tools for risk of osteoporotic fractures using data from population based electronic health records: retrospective cohort study. BMJ. 2017;356:i6755. doi:10.1136/bmj.i6755 28104610 PMC5244817

[20] Leslie WD, Majumdar SR, Morin SN, . FRAX for fracture prediction shorter and longer than 10 years: the Manitoba BMD registry. Osteoporos Int. 2017;28(9):2557-2564. doi:10.1007/s00198-017-4091-3 28593449

[21] Nguyen ND, Frost SA, Center JR, Eisman JA, Nguyen TV. Development of a nomogram for individualizing hip fracture risk in men and women. Osteoporos Int. 2007;18(8):1109-1117. doi:10.1007/s00198-007-0362-8 17370100

[22] Coviello V, Boggess M. Cumulative incidence estimation in the presence of competing risks. Stata J. 2004;4(2):103-112. doi:10.1177/1536867X0400400201

[23] Fine JP, Gray RJ. A proportional hazards model for the subdistribution of a competing risk. J Am Stat Assoc. 1999;94(446):496-509. doi:10.1080/01621459.1999.10474144

[24] Beaudoin C, Moore L, Gagné M, . Performance of predictive tools to identify individuals at risk of non-traumatic fracture: a systematic review, meta-analysis, and meta-regression. Osteoporos Int. 2019;30(4):721-740. doi:10.1007/s00198-019-04919-6 30877348

[25] Ensrud KE, Lui LY, Taylor BC, ; Study of Osteoporotic Fractures Research Group. A comparison of prediction models for fractures in older women: is more better? Arch Intern Med. 2009;169(22):2087-2094. doi:10.1001/archinternmed.2009.404 20008691 PMC2811407

[26] Ettinger B, Ensrud KE, Blackwell T, Curtis JR, Lapidus JA, Orwoll ES; Osteoporotic Fracture in Men (MrOS) Study Research Group. Performance of FRAX in a cohort of community-dwelling, ambulatory older men: the Osteoporotic Fractures in Men (MrOS) study. Osteoporos Int. 2013;24(4):1185-1193. doi:10.1007/s00198-012-2215-3 23179575 PMC3767034

[27] Fraser LA, Langsetmo L, Berger C, ; CaMos Research Group. Fracture prediction and calibration of a Canadian FRAX® tool: a population-based report from CaMos. Osteoporos Int. 2011;22(3):829-837. doi:10.1007/s00198-010-1465-1 21161508 PMC5101064

[28] Leslie WD, Lix LM, Johansson H, Oden A, McCloskey E, Kanis JA; Manitoba Bone Density Program. Independent clinical validation of a Canadian FRAX tool: fracture prediction and model calibration. J Bone Miner Res. 2010;25(11):2350-2358. doi:10.1002/jbmr.123 20499367

[29] Langsetmo L, Nguyen TV, Nguyen ND, ; Canadian Multicentre Osteoporosis Study Research Group. Independent external validation of nomograms for predicting risk of low-trauma fracture and hip fracture. CMAJ. 2011;183(2):E107-E114. doi:10.1503/cmaj.100458 21173069 PMC3033952

[30] Agarwal A, Leslie WD, Nguyen TV, Morin SN, Lix LM, Eisman JA. Predictive performance of the Garvan Fracture Risk Calculator: a registry-based cohort study. Osteoporos Int. 2022;33(3):541-548. doi:10.1007/s00198-021-06252-3 34839377

[31] Rubin KH, Friis-Holmberg T, Hermann AP, Abrahamsen B, Brixen K. Risk assessment tools to identify women with increased risk of osteoporotic fracture: complexity or simplicity? A systematic review. J Bone Miner Res. 2013;28(8):1701-1717. doi:10.1002/jbmr.1956 23592255

[32] Gourlay ML, Ritter VS, Fine JP, ; Osteoporotic Fractures in Men (MrOS) Study Group. Comparison of fracture risk assessment tools in older men without prior hip or spine fracture: the MrOS study. Arch Osteoporos. 2017;12(1):91. doi:10.1007/s11657-017-0389-1 29052793 PMC5695884

[33] Ensrud KE, Kats AM, Boyd CM, ; Study of Osteoporotic Fractures (SOF) Research Group. Association of disease definition, comorbidity burden, and prognosis with hip fracture probability among late-life women. JAMA Intern Med. 2019;179(8):1095-1103. doi:10.1001/jamainternmed.2019.0682 31206140 PMC6580441

[34] University of California at San Francisco. ePrognosis Calculators. Accessed December 20, 2023. https://eprognosis.ucsf.edu/calculators.php

[35] ClinRisk Ltd. QFracture-2016 risk calculator. 2019. Accessed December 20, 2023. https://qfracture.org/

[36] Livingstone SJ, Guthrie B, McMinn M, Eke C, Donnan PT, Morales DR. Derivation and validation of the CFracture competing risk fracture prediction tool compared with QFracture in older people and people with comorbidity: a population cohort study. Lancet Healthy Longev. 2023;4(1):e43-e53. doi:10.1016/S2666-7568(22)00290-2 36610448

