# Supplementary material for: Hip Fracture Risk Assessment Tools for Adults Aged 80 Years and Older
Source: JAMA Netw Open. 2024 Jun 28;7(6):e2418612. doi: 10.1001/jamanetworkopen.2024.18612 (PMC11214124; doi:10.1001/jamanetworkopen.2024.18612)
Supplement: Supplement 1. — eTable 1. Cohort Study Information eTable 2. Characteristics of Women by Cohort eTable 3. Characteristics of Men by Cohort eFigure 1. Participant Flow Diagram eFigure 2. Observed vs Predicted 5-Year Absolute Probability of Hip Fracture Stratified by Quintile of Predicted Risk for the Garvan Tool in Women and Men [file jamanetwopen-e2418612-s001.pdf]

Supplemental Online Content

Ensrud KE, Schousboe JT, Crandall CJ, et al. Hip fracture risk assessment tools for adults aged 80 years and older. *JAMA Netw Open*. 2024;7(6):e2418612. doi:10.1001/jamanetworkopen.2024.18612

eTable 1. Cohort Study Information

eTable 2. Characteristics of Women by Cohort

eTable 3. Characteristics of Men by Cohort

eFigure 1. Participant Flow Diagram

eFigure 2. Observed vs Predicted 5-Year Absolute Probability of Hip Fracture Stratified by Quintile of Predicted Risk for the Garvan tool in Women and Men

This supplemental material has been provided by the authors to give readers additional information about their work.

© 2024Ensrud KE et al. *JAMA Network Open.*

eTable 1. Cohort Study Information

Cohort[a]	Demographics	Geographic Region	Eligibility Criteria	Baseline Exam	Index Exam[b]
SOF	9,704 community-dwelling white women, and 662 community-dwelling black women	4 US sites (Baltimore, MD; Minneapolis, MN; Pittsburgh, PA; Portland, OR)	Women age 65 and older, ability to walk without the assistance of another person, and absence of bilateral hip replacement	1986-1988 white cohort; 1997-1998 black cohort	Year 10 (1997-1998) or Year 16 (2002-2004)
MrOS	5,994 community-dwelling men	6 US sites (Birmingham, AL; Minneapolis, MN; Palo Alto, CA; Pittsburgh, PA; Portland, OR; San Diego, CA)	Men age 65 and older, ability to walk without the assistance of another person, and absence of bilateral hip replacement	2000-2002	Year 5 (2005-2007), Year 7 (2007-2009) or Year 14 (2014-2016)
Health ABC	3,075 black and white community-dwelling men and women	2 US sites (Memphis, TN and Pittsburgh, PA)	Age 70-79 with no self-report of mobility difficulty or mobility disability	1997-1998	Year 3 (1999-2000), Year 5/6 (2002-2003), Year 8 (2004-2005) or Year 10 (2006-2007)

Study acronyms: SOF, Study of Osteoporotic Fractures; MrOS, Osteoporotic Fractures in Men Study; Health ABC, Health, Aging and Body Composition Study
[a]Institutional review boards at each participating site reviewed and approved the four cohort studies; all participants provided written informed consent
[b]Participants counted only once and included in analytical cohort at index examination when they first reached age ≥80 years

© 2024Ensrud KE et al. *JAMA Network Open.*

eTable 2. Characteristics of Women by Cohort

	Women	
Characteristic	SOF (n=4101)	Health ABC (n=805)
Age, years, mean (SD)	82.9 (2.9)	81.2 (0.9)
Race/ethnicity, n (%)		
Black	235 (5.7)	310 (38.5)
White	3,866 (94.3)	495 (61.5)
Weight, kg, mean (SD)	65.0 (12.4)	67.5 (13.8)
Body mass index, kg/m^2, mean (SD)	26.5 (4.7)	27.1 (5.3)
Number of fractures since age 50, n (%)		
0	1,904 (46.4)	509 (63.2)
1	725 (17.7)	249 (30.9)
2	910 (22.2)	40 (5.0)
≥3	562 (13.7)	7 (0.9)
Number of falls in past year, n (%)		
0	2,691 (65.6)	566 (70.3)
1	814 (19.8)	142 (17.6)
2	315 (7.7)	76 (9.4)
≥3	281 (6.9)	21 (2.6)
Parent fractured hip, n (%)[a]	494 (12.0)	0 (0)
Current smoker, n (%)	113 (2.8)	39 (4.8)
Oral glucocorticoid use, n (%)	137 (3.3)	28 (3.5)
Rheumatoid arthritis, n (%)	592 (14.4)	5 (0.6)
Alcohol intake ≥3 drinks/day, n (%)	37 (0.9)	1 (0.1)
Bisphosphonate use, n (%)	170 (5.3)	4 (0.5)
Femoral neck BMD T-score[b], mean (SD)	−2.0 (1.0)	−1.6 (1.0)
5-year probability (%) of hip fracture from FRAX w/BMD model, mean (SD)[a]	4.8 (4.9)	2.2 (2.1)
5-year probability (%) of hip fracture from Garvan w/BMD model, mean (SD)	18.7 (22.3)	6.9 (9.4)
5-year probability (%) of hip fracture from femoral neck BMD, mean (SD)	7.0 (5.4)	5.2 (4.3)

Abbreviations: BMD, bone mineral density; SD, standard deviation
[a]assuming no parental history of hip fracture for all Health ABC participants
[b]BMD T-scores were calculated using the National Health and Nutrition Examination Survey young white female reference database

© 2024Ensrud KE et al. *JAMA Network Open.*

eTable 3. Characteristics of Men by Cohort

Characteristic	Men	
	MrOS (n=3205)	Health ABC (n=779)
Age, years, mean (SD)	83.0 (2.9)	81.3 (1.0)
Race/ethnicity, n (%)		
Black	77 (2.4)	244 (31.3)
White	2,940 (91.7)	535 (68.7)
Other[a]	188 (5.9)	0 (0)
Weight, kg, mean (SD)	79.4 (12.5)	79.3 (13.2)
Body mass index, kg/m^2, mean (SD)	26.8 (3.7)	26.9 (4.0)
Number of fractures since age 50, n (%)		
0	2,242 (70.0)	607 (77.9)
1	626 (19.5)	165 (21.2)
2	207 (6.5)	7 (0.9)
≥3	130 (4.1)	0 (0)
Number of falls in past year, n (%)		
0	2,072 (64.6)	555 (71.2)
1	575 (17.9)	128 (16.4)
2	427 (13.3)	72 (9.2)
≥3	131 (4.1)	24 (3.1)
Parent fractured hip, n (%)[b]	431 (13.4)	0 (0)
Current smoker, n (%)	38 (1.2)	36 (4.6)
Oral glucocorticoid use, n (%)	89 (2.8)	19 (2.4)
Rheumatoid arthritis, n (%)	210 (6.6)	3 (0.4)
Alcohol intake ≥3 drinks/day, n (%)	132 (4.1)	13 (1.7)
Bisphosphonate use, n (%)	170 (5.3)	4 (0.5)
Femoral neck BMD T-score[c], mean (SD)	−0.9 (1.1)	−0.8 (1.2)
5-year probability (%) of hip fracture from FRAX w/BMD model, mean (SD)[b]	2.2 (2.4)	1.2 (1.0)
5-year probability (%) of hip fracture from Garvan w/BMD model, mean (SD)	6.1 (9.6)	3.6 (5.1)
5-year probability (%) of hip fracture from femoral neck BMD, mean (SD)	3.1 (3.4)	3.1 (3.5)

Abbreviations: BMD, bone mineral density; SD, standard deviation
[a]Asian, Hispanic, Latino, Native American or Hawaiian/Pacific Islander
[b]assuming no parental history of hip fracture for all Health ABC participants
[c]BMD T-scores were calculated using the National Health and Nutrition Examination Survey young white female reference database

© 2024Ensrud KE et al. *JAMA Network Open.*

eFigure 1. Participant Flow Diagram

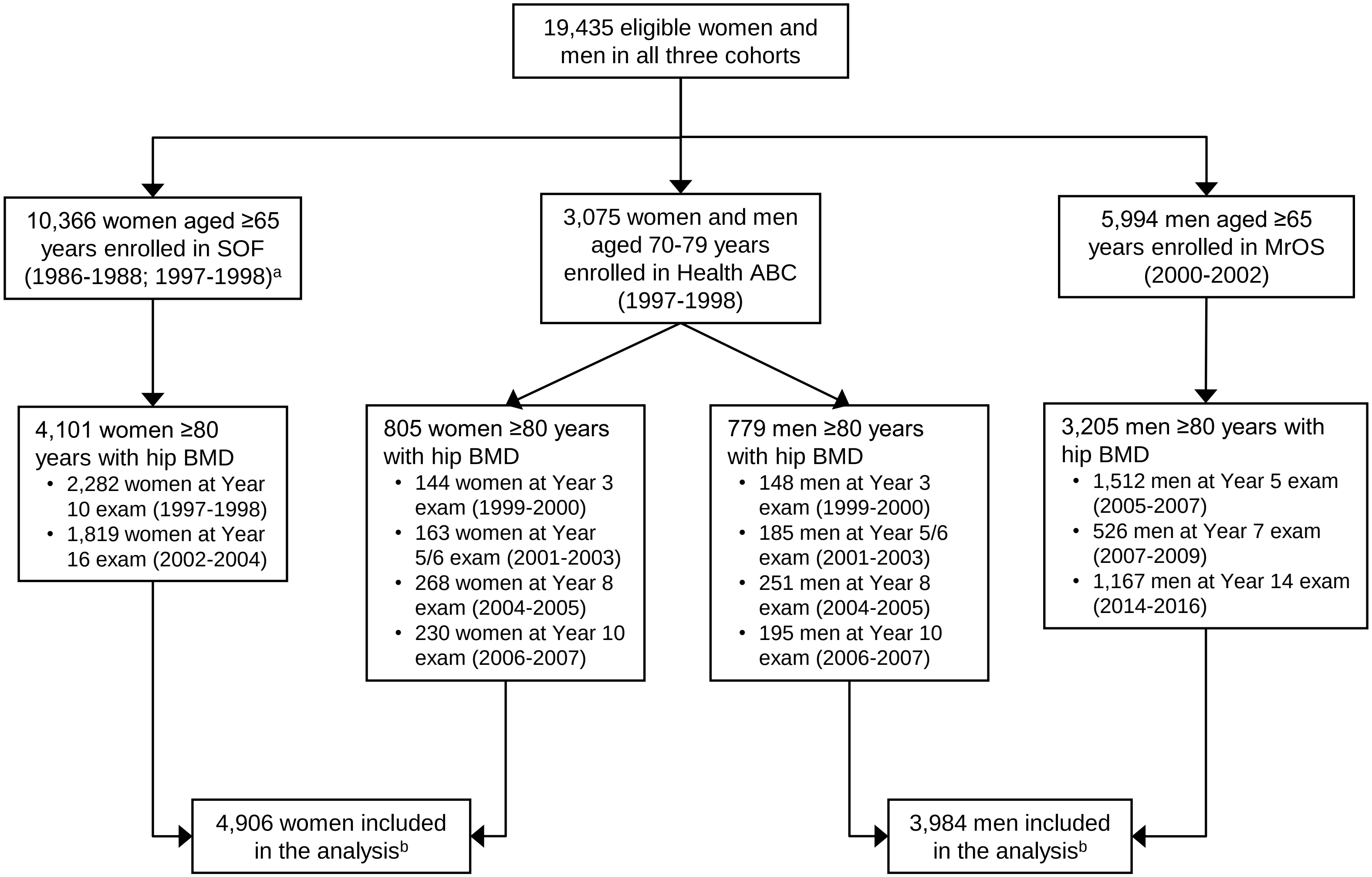

[a]9,704 community-dwelling white women were enrolled from 1986-1988, and 662 community-dwelling black women were enrolled from 1997-1998
[b]Participants counted only once and included in analytical cohort at index examination when they first reached age ≥80 years

© 2024Ensrud KE et al. *JAMA Network Open.*

eFigure 2. Observed[a] vs Predicted 5-Year Absolute Probability of Hip Fracture Stratified by Quintile of Predicted Risk for the Garvan tool in Women and Men

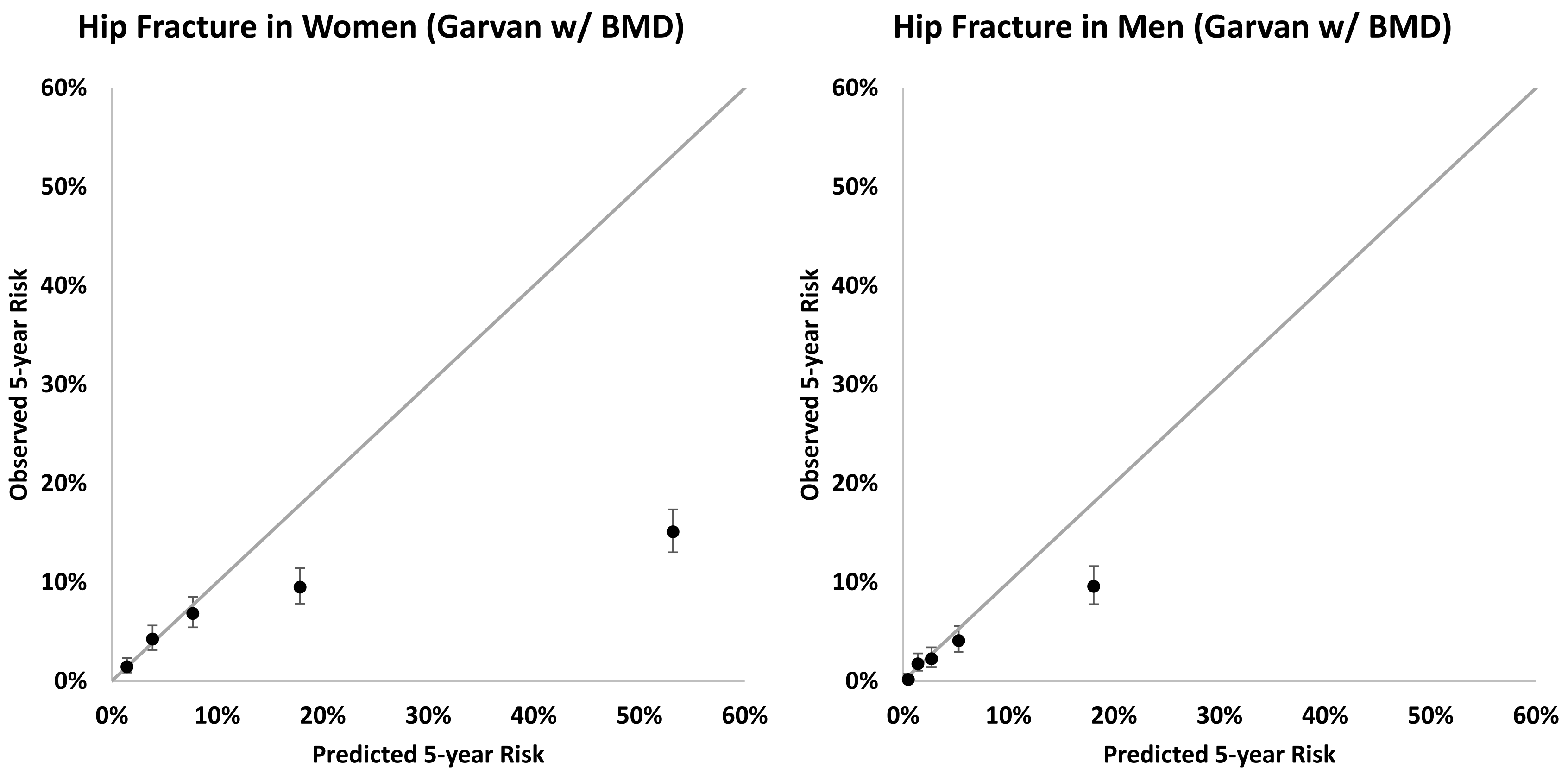

[a]observed probability of hip fracture was calculated using Kaplan-Meier method

© 2024Ensrud KE et al. *JAMA Network Open.*
