# Supplementary material for: Hip Fracture Risk Assessment Tools for Adults Aged 80 Years and Older
Source: JAMA Netw Open. 2024 Jun 28;7(6):e2418612. doi: 10.1001/jamanetworkopen.2024.18612 (PMC11214124; doi:10.1001/jamanetworkopen.2024.18612)
Supplement: Supplement 2. — Data Sharing Statement [file jamanetwopen-e2418612-s002.pdf]

Data Sharing Statement

Ensrud. Hip Fracture Risk Assessment Tools for Adults Aged 80 Years and Older. *JAMA Netw Open*. Published June 26, 2024. doi:10.1001/jamanetworkopen.2024.18612

Data
Data available: Yes
Data types: Deidentified participant data, Data dictionary
How to access data: SOF data is available to the public via the 'SOF Online' website (https://sofonline.ucsf.edu/). MrOS data is available to the public via the 'MrOS Online' website (https://mrosonline.ucsf.edu/). Health ABC data is available to the public via the 'Health ABC Study' website (https://healthabc.nia.nih.gov/).
When available: With publication

Supporting Documents
Document types: None

Additional Information
Who can access the data: Anyone
Types of analyses: For any purpose
Mechanisms of data availability: registered account with data use agreement
